# Carbon innumeracy

**DOI:** 10.1371/journal.pone.0196282

**Published:** 2018-05-03

**Authors:** Amir Grinstein, Evan Kodra, Stone Chen, Seth Sheldon, Ory Zik

**Affiliations:** 1 Associate Professor of Marketing, D’Amore-McKim School of Business, Northeastern University, Boston, MA, United States of America; 2 Associate Professor of Marketing, School of Economics and Business Administration, VU Amsterdam, The Netherlands; 3 risQ Inc., Cambridge, MA, United States of America; 4 Sheldon Data, Athens, OH, United States of America; 5 Oryzik.com, Brookline, MA, United States of America; University of Illinois, UNITED STATES

## Abstract

Individuals must have a quantitative understanding of the carbon footprint tied to their everyday decisions to make efficient sustainable decisions. We report research of the innumeracy of individuals as it relates to their carbon footprint. In three studies that varied in terms of scale and sample, respondents estimate the quantity of CO_2_ released when combusting a gallon of gasoline in comparison to several well-known metrics including food calories and travel distance. Consistently, respondents estimated the quantity of CO_2_ from gasoline compared to other metrics with significantly less accuracy while exhibiting a tendency to underestimate CO_2_. Such relative absence of carbon numeracy of even a basic consumption habit may limit the effectiveness of environmental policies and campaigns aimed at changing individual behavior. We discuss several caveats as well as opportunities for policy design that could aid the improvement of people’s quantitative understanding of their carbon footprint.

## Introduction

While political action is already underway in multiple countries in response to urgent calls for reductions in greenhouse gas emissions such as the widely discussed carbon footprint [1,2,3] recent political developments in the U.S.[4] create significant uncertainty regarding policies to combat climate change. Thus, as 58% of Americans worry about climate change [5] and since consumers and households significantly contribute to greenhouse gas emissions, it is becoming even more important that part of the burden be carried at the individual level [6,7]. However, individuals are not clear about the relative contribution to greenhouse gas emissions of their behavior [8]. Metrics such as carbon footprint remain unclear, intangible byproducts of day-to-day consumer activities, as well as activities that connect to the consumer through complex material and energy supply chains. Such metrics are largely irrelevant for personal decisions by many individuals.

In this research, we measure the degree to which people’s carbon numeracy is tied to their consumption behaviors. We define carbon numeracy as one’s ability to approximate a correct value of one’s carbon footprint without resorting to an explicit calculation. Familiar examples are food calories and ambient temperature, where people can interpret a quantitative signal (e.g., the temperature) to make decisions (e.g., clothing), without performing a calculation [9]. The motivation for our work is the notion that when the belief system and assumptions that guide climate-related decisions are grounded in physical reality (e.g., in the form of labeling), decisions are likely to be more effective [10,11]. This requires numeracy. As far as we are aware, research thus far has not attempted to measure the degree of carbon numeracy of people’s consumption behavior. This paper’s main goal is therefore to be the first step toward addressing this knowledge gap. Specifically, through three studies, this research measures and analyzes respondents’ ability to estimate the direct carbon emissions of driving a car due to gasoline consumption. We present a simple test that involves comparing the ability to estimate the carbon impact of combusting a gallon of gasoline to other daily metrics, such as food calories in a gallon of whole milk and travel distance. We select gasoline consumption and a gallon of gas as they reflect one of the simplest and most consistent activities and measures in the daily life of many people. We believe that the analogy to food calories is especially useful since calories have been extensively used as a nutrition metric for battling obesity, a role analogous to carbon footprint in mitigating climate change [12]. First, we report two small-scale surveys of the general population and university students (studies 1a and 1b, respectively). Next, we present a large-scale survey that corroborates the initial results among the general population and addresses multiple constraints of the small-scale studies (study 2).

The rest of this paper is structured as follows. First, we review relevant literature on carbon numeracy and human decision making. Then we report findings and their implications and caveats. Finally, we discuss theoretical and policy implications of this work.

Better understanding of the nature of people’s estimation of the carbon footprints of their behaviors will help policymakers to strengthen greenhouse gas reduction policies and create more effective communication that is aimed at changing consumer behavior [9,13,14].

### Numeracy, estimation error and bias: Theoretical background and predictions

People’s numerical understanding of the environmental consequences of their consumption behavior is often viewed as critical for more responsible consumption behavior [15]. Essentially, numeracy creates a context for behavior (i.e., “is my environmental impact low or high?”). Such numerical understanding when grounded in physical reality (e.g., in the form of labeling) can transform environmental values into actions [10,11]. For example, it can lead to an effective behavioral change that can promote more energy savings that offer both environmental and economic benefits [16].

However, the numeracy of individuals as it pertains to decision-making domains is prone to error [17]. In the specific case of understanding the carbon footprint consequences of one’s consumption behavior, a recent study [9] suggests that people’s ability to quantify and capture the impact of their actions on the environment is very limited. The study argues that when making decisions, people typically rely on common systems and quantitative signals (e.g., price, temperature, food calories) that are consistent (i.e., accurate) and that are often practiced. Often, people’s familiarity and suitable approximations of these metrics–their consistency and the fact that they are often practiced–are used to support decisions [17,18]. Such numeracy about sustainability is, for the most part, apparently absent. In a recent survey of UK consumers, only 14% stated that they knew their own personal carbon footprint and 89% thought carbon labeling is confusing [19].

Lack of numeracy about carbon footprint is tied to consumption decisions and has two consequences. First, lack of intuition at the individual level is likely to be translated to a highly variable distribution of perceptions and attitudes among people at the aggregate level [20,21]. This would require policymakers to design multiple interventions that are aimed at different consumer groups and invest more in educating individuals. Second, many people will tend to underestimate the carbon implications of their consumption choices. In a survey of American households, Attari et al. [22] found that the average respondent underestimated potential usage of energy and water by a factor of nearly three. Similarly, Gardner and Stern [6] reviewed multiple situations where consumers underestimate energy usage and its consequences. A possible explanation is that people tend to view themselves and their behavior in a positive light [23], underestimating the potential negative consequences of their undesirable behavior [24,25]. This may be especially pronounced in domains related to environmental issues where moral norms play a role [26].

Within the context of this research, we predict that innumeracy would be evidenced by carbon estimations with very large distribution (i.e., error). We further predict that the distribution will be biased downward, demonstrating clear underestimation of carbon footprint. These errors and biases in carbon estimation are expected to be much higher than estimations that suffer less from innumeracy such as food calories or travel distance.

## Materials and methods

Ben-Gurion University’s Guilford Glazer School of Business’ Human Subjects Research Committee has approved the project “Carbon Illiteracy: Demonstrating a Prevalent Lack of Quantitative Environmental Intuition” (AG_05022015). Written informed consent was obtained from participants in all our studies.

### Study plan

We designed and performed three survey-based studies to analyze the current state of people’s innumeracy about the carbon footprint associated with using one gallon of gasoline, as compared to other metrics that are ubiquitous in daily decisions and consumption (i.e., food calories, distances) or that are less ubiquitous (i.e., car weight). Further, the choice of one gallon is motivated by the desire to compare responses for equivalent volumes using a unit that is most familiar to consumers in the United States, where the studies were conducted. The studies, complement one another in several ways (a summary of the key characteristics of the studies appear in Table 1). First, they involve different participants–from the general population as well as students. A key reason to conduct the study among students (i.e., not only among the general population) is the fact that the former are considered more pro-environmental [27], which make their study a conservative test of our predictions. Second, the studies involve online and in-person data collection efforts to control for the possible influence of external information on respondents’ estimations. Third, the studies involve different comparisons to carbon footprint estimation (e.g., comparison with food calories). Fourth, while the two initial studies involve relatively limited sample sizes (N<200), the third survey targeted 961 participants. Finally, the large-scale study addressed various potential methodological concerns of the initial studies, as discussed below.

**Table 1:** The key characteristics of the three studies.

Study	Sample size	Population	Data collection approach	Key measures/comparisons
1a	175	General population	Online	-CO_2_ of 1 gallon of gasoline -Calories in 1 gallon of whole milk
1b	100	Students	Face-to-face	-CO_2_ of 1 gallon of gasoline -Calories in 1 gallon of whole milk -Travel distance from NYC to LA
2	961	General population	Online	-CO_2_ of 1 gallon of gasoline -Calories in 1 gallon of whole milk -Travel distance from NYC to LA -Weight of an average family car

S1 Text (Methodological Appendix A) provides the detailed surveys for each of the three studies.

### Analytical approach

In each study, every respondents’ estimates were divided by the actual value to obtain the following estimation ratios (study 1a: CO_2_ and milk, study 1b: CO_2_, milk and distance, study 2: CO_2_, milk, distance and car weight):

$$R_{c}=\frac{est.{CO}_{2}}{true\;{CO}_{2}},R_{m}=\frac{est.calories}{true\;calories},R_{w}=\frac{est.weight}{true\;weight},and\;R_{d}=\frac{est\;distance}{true\;distance}.$$

From these ratios, two metrics are defined and calculated: estimation error and estimation bias.

We define estimation error as the absolute value of the log_10_ transformed estimation ratios:

$$|\log_{10}\left(R_{c}\right)|,|\log_{10}\left(R_{m}\right)|,|\log_{10}\left(R_{w}\right)|,\;and\;|\log_{10}\left(R_{d})\right|.$$

This definition is justified as follows: a perfect estimate will result in an estimation error of zero, |log_10_(1)| = 0, and any underestimate or overestimate will result in a positive number. The log_10_ transformation aims to help reduce the influence of large outliers in subsequent analysis. Estimation error defined here provides a measure of the innumeracy of a given respondent regarding any of the studied metrics (CO_2_, milk calories, distance or car weight).

Estimation bias is defined and calculated by taking the log_10_ of the ratios: log_10_(*R_c_*),log_10_(*R_m_*),log_10_(*R_w_*),log_10_(*R_d_*), but without taking their absolute value. These log_10-_transformed ratios allow us to treat underestimates and overestimates symmetrically. For example, an estimation ratio of 10 (ten times higher than actual) becomes log_10_(10) = 1 and a ratio of 0.1 (one-tenth of actual) becomes log_10_(0.1) = -1. These values are reported in S1 Text (Methodological Appendix B). The mean biases and associated 95% confidence intervals are reported as a percentage of the actual value. Mean biases and their associated confidence intervals are computed by converting back to original units. For example, a mean bias of log_10_(0.1) = -1 is reported 10^−1^ * 100% = 10% of the actual value.

In all studies, estimation error and bias are both separately analyzed using a mixed effects ANOVA model. To determine if there is a difference in the mean estimation error, a mixed-effects ANOVA model was used. The estimation error was fitted with the factor (CO_2_, milk calories, distance or car weight) as a fixed effect and the subject as a random effect. See S1 Text (Methodological Appendix B) for details.

### Study 1a: Online study of the general population

#### Respondents

The first study involved a survey of N = 175 participants (73 women, mean age = 35) from the U.S. They were recruited online using Amazon Mechanical Turk–an online tool used widely to crowd source data from the general population. Specifically, Amazon Mechanical Turk is a web service that coordinates the supply and demand of different tasks (including academic studies) that require human intelligence to complete. It is an online labor market where employees (called workers) are recruited by employers (called requesters) for the execution of the tasks in exchange for a wage (called a reward) while both workers and requesters remain anonymous [28].

The initial sample for the study involved 208 participants. However, we excluded from the analysis 33 participants who did not follow the instructions: 22 assessed CO_2_ estimation across all measurement units when asked to estimate using only a single unit and an additional 11 did not estimate both CO_2_ and food calories. We note that removing the 33 respondents does not qualitatively change the nature of the core findings. Data set can be found in S2 Table.

#### Procedure

We asked respondents to use only their intuition and estimate 1) the amount of CO_2_ emitted by burning one gallon of gasoline in their car, and 2) the food calories contained in 1 gallon of standard dairy milk. In the case of CO_2_ emissions, we allowed for answers in U.S. customary units (e.g., pounds) and metric units (e.g., kilograms) to make estimation easy across American and non-American participants. The actual values are approximately 9 kg of CO_2_ [29], and 2,400 Calories, respectively. Specifically, we asked: “How much CO2 do you think is emitted in the production/consumption of 1 gallon of standard gasoline in your car?” and, “How much calories do you think are contained in 1 gallon of standard dairy milk?”

For each respondent, the sequence of these questions was randomized to minimize any order effects. We also asked about general environmental involvement, based on the Consumer Involvement Scale [30]. The scale is a five-point semantic differential scale that consists of five adjectives relating to the statement–“I find environmental and ecological issues…”: “not important/important,” “not essential/essential,” “not valuable/valuable,” “not interesting/interesting,” and “not significant/significant.” Finally, we asked respondents about potential educational background that might influence their results (e.g., background in sustainability, chemistry or physics).

#### Analysis and results

The main finding is that mean estimation error in the case of CO_2_ is significantly higher than for calories as determined by mixed-effects ANOVA (see Tables 2 and 3). Specifically, holding all else constant, the mean estimation error for calories in milk is 0.450 (95% confidence interval (CI) of 0.38–0.517) and 1.145 for CO_2_ from gasoline (95% CI of 0.994–1.295). Secondly, holding all else constant, there is a tendency to underestimate the amount of CO_2_ from gasoline with a mean bias of -0.750, which translates to 17.7% of the actual value (95% CI of 11%-28%), similar to that for calories in milk, which holding all else constant has a mean 44% of the actual value (95% CI of 37–53%).

**Table 2:** Mixed-effects ANOVA outputs for estimation error.

	Factor	Mean	Std. Error	DF	t-value	p-value
Study 1a	CO_2_ in gasoline	1.145	0.059	174	19.269	<0.000
	Calories	-0.695	0.082	174	-8.494	<0.000
Study 1b	CO_2_ in gasoline	2.364	0.100	198	23.743	<0.000
	Distance	-1.622	0.141	198	-11.522	<0.000
	Calories	-1.184	0.141	198	-8.412	<0.000
Study 2	CO_2_ in gasoline	0.867	0.019	2880	45.933	<0.000
	Distance	-0.525	0.026	2880	-20.338	<0.000
	Calories	-0.169	0.026	2880	-6.564	<0.000
	Car Weight	-0.460	0.026	2880	-17.808	<0.000

**Table 3:** Summaries of mean estimation error and bias with 95% CIs (all experiments).

	Factor	Mean	Std. Error	Lower Bound	Upper Bound
Study 1a	CO_2_ in gasoline (bias)	-0.75	0.1	-0.95	-0.55
	Calories	-0.35	0.04	-0.43	-0.27
	CO_2_ in gasoline (error)	1.15	0.08	0.99	1.3
	Calories	0.45	0.03	0.38	0.52
Study 1b	CO_2_ in gasoline (bias)	-2.36	0.16	-2.66	-2.05
	Distance	0.4	0.09	0.22	0.58
	Calories	-1.18	0.04	-1.26	-1.09
	CO_2_ in gasoline (error)	2.36	0.15	2.06	2.67
	Distance	0.74	0.07	0.61	0.87
	Calories	1.18	0.04	1.1	1.26
Study 2	CO_2_ in gasoline (bias)	-0.58	0.03	-0.64	-0.51
	Distance	0.12	0.02	0.09	0.16
	Calories	-0.62	0.02	-0.66	-0.58
	Car weight	-0.27	0.02	-0.3	-0.23
	CO_2_ in gasoline (error)	0.87	0.02	0.82	0.91
	Distance	0.34	0.02	0.31	0.37
	Calories	0.7	0.02	0.66	0.74
	Car weight	0.41	0.02	0.38	0.44

Importance of environmental issues and potentially relevant educational background were not factors that had a significant effect on either estimation error or bias. We also report in S1 Table the results in percent bias of the real value for all studies.

### Study 1b: Face-to-face study of the student population

In study 1b, we sought to further explore the above observations but also to extend our analysis in three ways. First, a concern with study 1a is that participants could have consulted external information during estimation (e.g., online carbon footprint calculators), although they were explicitly instructed not to. Thus, study 1b adopts an in-person moderated procedure with no Internet access that eliminates this concern. Second, study 1a involved a general population sample. In study 1b, we examine whether the findings hold with a sample of university students, potentially characterized by higher levels of environmental awareness [31]. Third, in study 1a we compared estimates of CO_2_ in standard gasoline to those of calories in whole milk. One may argue that respondents could have especially high interest in developing a strong numeracy about calories because they have a direct personal impact on one’s health. Therefore, in study 1b, apart from a comparison to calories in a gallon of milk, we also prompt participants to estimate a less personal, but nevertheless practiced, metric of travel distance. Travel distances, especially long ones, are not likely to be familiar to most people, but we might expect respondents to have relatively higher numeracy about distance in general compared to carbon footprint.

#### Respondents

One hundred student participants were recruited by a research assistant on the campus of a large American university. Our sample is comprised of students from 22 different majors, attesting to the academic diversity of the students participating in this study. Data set can be found in S3 Table.

#### Procedure

As in study 1a, we first asked the respondents to use only their intuition and estimate both the amount of CO_2_ emitted by burning one gallon of gasoline in their car in one of the following measurement units–pounds, kilograms, grams or metric tons as well as the number of calories in one gallon of whole milk. We used the same questions as in study 1a. The students were then asked to estimate the travel distance from Los Angeles to New York City in miles or kilometers (approximately 3900 km). We used the same questionnaires as in study 1a (however, gender and age information were not collected) including one item from Mittal’s [30] importance of environmental issues scale (“I find environmental and ecological issues… not important/important”) and a question about the student’s major at the university.

#### Analysis and results

As a starting point, we tested the assumption that the student population from study 1b would view environmental topics as more important than the general population studied in study 1a. A two-sided t-test suggests that the student population rates environmental importance (EI) significantly higher on average. The mean estimate of EI for study 1b’s student respondents was 4.46, while the EI for the respondents in study 1a was 4.15 (t = 3.05, p = 0.003).

**Fig 1:**
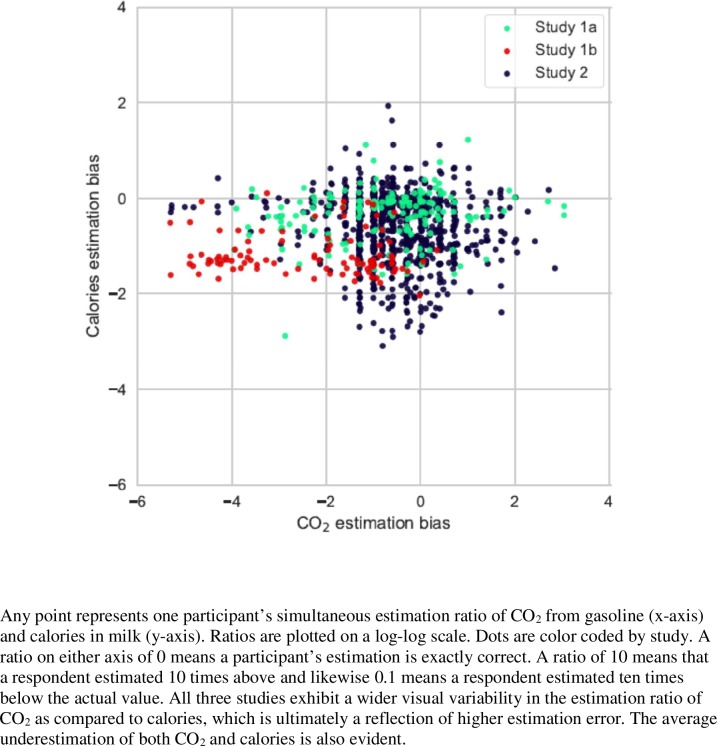
Results of studies 1a, 1b, and 2 –carbon versus calorie estimations.

Similar to the first study, holding all else constant, we find significantly larger estimation error for CO_2_ than calories or estimates of distance from Los Angeles to New York as determined by the mixed-effects ANOVA model (p-value < 0.001; findings are reported in Tables 1 and 2, as well as Fig 1).

The mean estimation error of CO_2_ emission from burning gasoline is 2.364 (95% CI of 2.062–2.665 and for calories in whole milk is 1.179 (95% CI of 1.095–1.264), corroborating results from Study 1a. Further, the mean estimation error in kilometers to travel from Los Angeles to New York City is 0.742 (95% CI of 0.614–0.869).

As with study 1a, there is a significant underestimation tendency for the amount of CO_2_ released from combusted gasoline, with a mean estimation bias of 0.44% of the actual value (95% CI of 0.22%-0.89%) as well as calories in milk with a mean estimation bias of 6.7% of the actual value (95% CI of 5.5%-8.1%).

Estimates of carbon from gasoline vary significantly more than estimates of travel distance. Interestingly though, in contrast to CO_2_ and calories, there is a tendency to overestimate distance with a mean estimation bias of 250% of the actual value (95% CI of 166%-377%). This finding is in line with the notion that people overestimate objects or realities that lack visibility like travel distances [21].

### Study 2: Large-scale online study of the general population

#### Respondents

Study 2 involved a survey of N = 961 participants (496 women, mean age~ = 42) from the U.S. As with study 1a, they were recruited online using Amazon Mechanical Turk. The initial sample for the study involved 1,046 participants. However, we excluded from the analysis 70 participants that did not put an appropriate estimate (e.g., “I don't know,” “little,” “5 percent”). An additional 15 were removed for estimating 0 for either pounds of CO2, calories of milk, distance between NY and LA, and weight of a car. Data set can be found in S4 Table.

#### Procedure

We first asked respondents to use only their intuition and estimate both the amount of CO_2_ emitted by burning one gallon of gasoline in a motor vehicle (to avoid confusion we only asked participants to use pounds; “What quantity of CO2 do you think is emitted by consuming 1 gallon of standard gasoline when driving a motor vehicle?”), as well as the number of calories in one gallon of whole milk (in calories; “How many calories do you think are contained in 1 gallon of whole milk?”), the distance between Los Angeles and New York City (in miles; “What do you think is the distance between Los Angeles and New York (coast-to-coast)?”) and another estimation that unlike the previous ones has no direct personal relevance to day-to-day lives of consumers and is less practiced: the weight of an average family car (in pounds; 1,850 kg [32]; “What do you think is the weight of an average family car in the U.S.?”). The estimation requests were presented in a random order.

We then included a single item that checked participants’ attention (“You need to check the third answer in the following question”). All participants successfully passed the attention check. To examine if results depend on pro-environmental attitudes, we included a single item about environmental importance (based on Mittal [30]). Finally, to examine if results depend on political orientation (conservative vs. liberal) we included an additional set of items [33,34]. Demographic questions concluded the survey; these can be found in detail in S1 Text (Methodological Appendix A).

#### Analysis and results

As with the first two studies, the difference in mean estimation error was determined with a mixed-effects ANOVA model. The model determined that mean CO_2_ estimation error was significantly larger than mean estimation error of milk, distance and car weight (p-values all < 0.001; findings are reported in Tables 1 and 2, as well as Fig 1).

From largest to smallest, the mean estimation error for each was: 0.867 for CO_2_ from gasoline, 0.698 for calories in milk, 0.408 for car weight, and 0.342 for distance from Los Angeles to New York City.

As with studies 1a and 1b, there is a tendency to underestimate the amount of CO_2_ from a gallon of gasoline with an estimate of 27% of the actual value (95% CI of 23%-31%) and calories in a gallon of milk with a mean estimate of 24% of the actual value (95% CI of 22%-26%). The weight of a car also tends to be underestimated with a mean estimate of 55% of the actual value (95% CI of 50%-59%). Again, distance in miles from Los Angeles to New York City tends to be overestimated with a mean estimate of 134% of the actual value (95% CI of 122%-145%).

**Fig 2:**
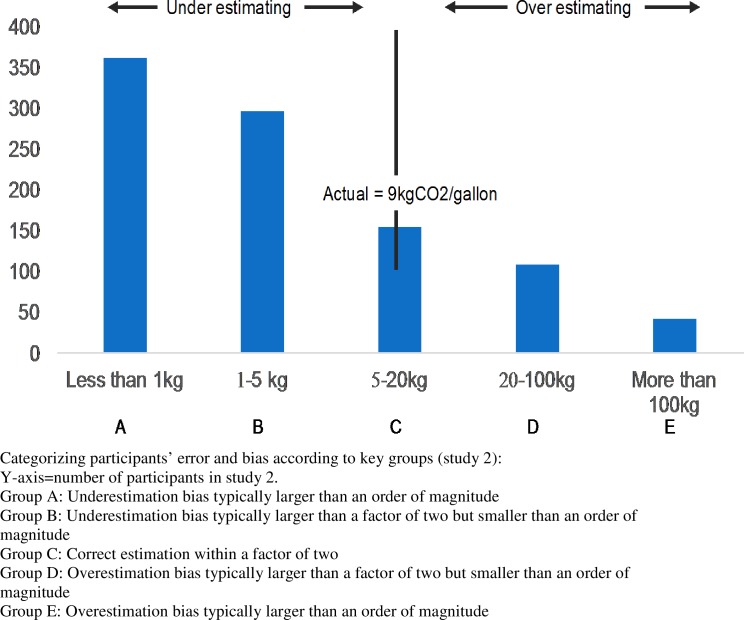
A simplified visualization of study 2’s results.

Fig 2 describes the findings of study 2 in a visual, more simplified manner.

## Discussion and conclusions

Results from three studies provide evidence supporting the hypothesis that people have a higher degree of carbon innumeracy tied to consumption than they have for other more commonly practiced and understood metrics like calories and distances (and even more than less practiced metrics like the weight of an average family car). We define estimation error and bias measures and analyze those through mixed effect ANOVA models. Results show that on average respondents consistently estimate the amount of CO_2_ from one gallon of standard gasoline significantly less accurately than they are able to estimate the number of calories in a gallon of whole milk, the travel distance from Los Angeles to New York City, or the weight of an average family car. The higher estimation error may not come as a surprise since, unlike food calories or distance that are practiced by individuals in everyday life [17], measurements of CO_2_ are not part of everyday decisions. This innumeracy is also evident in our final survey where CO_2_ estimates fall short even when compared to respondents’ ability to estimate the weight of an average family car, even though car weight is not a measure that is practiced commonly.

Overall, the results provide evidence that, despite increasing public awareness and discourse about climate change, environmental impact, and carbon footprinting [5,6] people cannot estimate their carbon footprint associated with the common practice of driving a motor vehicle. Further, even respondent samples that on average are more pro-environmental (students in in study 1b) tend to exhibit lower carbon numeracy compared to calories and distance.

We also consistently find a statistically significant tendency to underestimate the amount of CO_2_ from combusting gasoline. This bias is directionally consistent with prior research of American households, in which respondents underestimated potential usage of energy by a factor of nearly three [22] or four [6]. This underestimation bias could be explained by people’s tendency to underestimate the potential negative consequences of their undesirable behavior [24,25].

Remaining questions include whether general carbon innumeracy also characterizes experts and policymakers, not only laymen, and to what extent their carbon numeracy is correlated with the climate efficacy of their decisions. This may be especially valuable in the context of battling climate change as policymakers and sustainability experts are the ones designing policies and campaigns that aim to make a change [6,16]. Future research should address this question rigorously, for example, surveying environmentally savvy participants such as students enrolled in a sustainability programs or environmental consultants.

### Conclusions and policy implications

We have provided multiple cases of heuristic empirical evidence for carbon innumeracy, exemplified using a gallon of gasoline as the simplest way of capturing the consumption habit of using a car.

Our findings point to the need by policymakers, responsible marketers, NGOs, and academic scholars to remedy the ways in which individual carbon and other environmental footprints are quantified and communicated. One way to interpret Kahneman’s work [17] is that numeracy (and broadly quantitative intuition) will require both consistency and practice. Enhancing carbon numeracy may be achieved by multiple, non-mutually exclusive approaches, which we discuss next.

As a starting point, it is critical that the available carbon information is consistent. For instance, given the complex sources of energy in the U.S. (e.g., coal, solar, hydro, wind) it is not often clear how efficient would be an electric car [35]. Practicing an inconsistent signal will be less efficient in reducing the estimation error and will not contribute to carbon numeracy. Further efforts by scientists and policy makers should focus on the consistency of carbon footprint calculations in various systems, especially those that involve complex, location dependent systems such as the emissions tied to electricity consumption [36].

As far as practicing carbon footprint, the traditional approach is to create more knowledge about, and awareness for, carbon footprint and its meaning. For example, creating an educational campaign that establishes carbon as a metric for environmental impact. Environmental campaigns however are costly and there is limited evidence as to their effectiveness [37]. A different way to implement this approach is to integrate carbon footprint information in consumers’ physical space. For example, providing carbon footprint information on consumer goods packaging. Prior research suggests this can influence consumers’ decision making significantly [10,11]. A related example is to implement at various instant feedback mechanisms (e.g., energy use indoor digital meters, mobile apps). A prerequisite for an effective implementation of any policy involving product labeling or other awareness building mechanisms is carbon numeracy. Our work exposes the gap that we should bridge.

A second approach may be to establish a more intuitive alternative metric than carbon footprint, based on preexisting numeracy that rigorously represents carbon emissions as a proxy. For example, one can conceive of augmenting carbon footprint with “climate points,” “energy points,” or equivalencies that reflect negative monetary externalities that are equal to or associated with a kilogram of CO_2_ or the embodied energy of a gallon of gasoline, respectively. This concept is similar to the way Weight Watchers induced dietary behavior using a points system to augment calories [38]. While the use of calories leads to various biases [18,39] and it may be more effective for some populations [40], overall there is a positive, albeit modest, positive effect of calorie food labeling on food consumption [41]. Essentially, food calories and labeling can more broadly work by filling an information gap that may have an effect on subsequent decision making [40].

The advantage of the latter approach is having a metric that accounts for carbon but is less prone to estimation errors and not as dependent on expensive educational campaigns that are limited in effectiveness. This approach might be able to establish an environmental parallel of “calories” to quantify the impact of resource consumption of a given product or service. Such a metric will be especially valuable as it will enable people to make better environmental decisions when comparing alternative behaviors with environmental impact that carries different metrics (e.g., installing solar panels vs. eating local food). One promotional approach expected to be relatively low cost and effective would be to display this type of metric or environmental informatics in the general marketplace (e.g., near gasoline prices at gas stations; [42]).

### Limitations and future research

We highlight several caveats that can be addressed by future research. First, gasoline and milk were selected because of their simplicity, ubiquity, consistency and similarity in price per volume, but we did not independently determine whether gasoline and milk are representative of decisions that involve carbon and calories, respectively.

In addition, considering the issue of consistency, we chose a gallon of gas as it is perhaps the simplest and most consistent example. Thus, across our studies respondents did not have to estimate the carbon footprints tied to other products or services that vary in composition. Other consumption staples such as food, water, and electricity indirectly produce greenhouse gas consequences that vary by space and time, and are thus less suggestive to our study but should be included in future studies.

Second, in the current study we examined respondents’ absolute intuitive knowledge about carbon footprint strictly as it relates to standard gasoline. Future work may study relative knowledge on carbon footprints, for instance, by asking people to rank by impacts of various consumption behaviors from low to high on their estimated carbon footprint, as this may be highly relevant to people’s decision making.

Third, we should point out that our measurement approach was deliberately U.S.-centric (using gallons for example), but can be expanded to other countries. We nevertheless make sure to report our findings in metric units to enable generalizability.

Fourth, the question about CO_2_ in surveys 1a and 1b asks about the “production/consumption” of 1 gallon of standard gasoline in one’s car. Initially, we considered including the indirect carbon emissions associated with the gallon of gasoline itself (i.e., crude oil extraction, oil refining, prior to combustion by the driver), but ultimately decided to focus on direct emissions for simplicity. Unfortunately, the artifact was not caught before the surveys were administered. Given the context and low probability that the respondents would understand the phrase to be asking about emissions outside of the boundary of a car engine, and the consistent in results with survey 2, in which the error was addressed, we think that the impact on the results is minimal.

Finally, future work may find it useful to study more rigorously whether estimation error and bias also characterize sustainability experts, not only laymen.

## Supporting information

S1 Table The results in percent bias of the real value for all studies. (XLSX)

S2 Table Data_study1a. (XLSX)

S3 Table Data_study1b. (XLSX)

S4 Table Data_study2. (XLSX)

S1 Text Methodological Appendix: A, B.
(DOCX)
