# Supplementary material for: Carbon innumeracy
Source: PLoS One. 2018 May 3;13(5):e0196282. doi: 10.1371/journal.pone.0196282 (PMC5933710; doi:10.1371/journal.pone.0196282)
Supplement: S1 Text — Methodological Appendix: A, B. (DOCX) [file pone.0196282.s005.docx]

**S1 Text.** **Methodological Appendix: A, B**

**Methodological Appendix A: Survey Questions**

**Study 1a**

Please read the following question and answer in the space below. Please ONLY use your intuition and not external sources of information. How much CO2 do you think is emitted in the production/consumption of 1 gallon of standard gasoline in your car? ("carbon footprint"; please use only ONE of the following measures when providing your answer: either grams, kilograms, pounds or tons)

grams _____

kilograms _____

pounds ______

tons _______

Please read the following question and answer in the space below. Please ONLY use your intuition and not external sources of information. How much CO2 do you think is emitted in the production/consumption of 1 gallon of standard dairy milk? ("carbon footprint"; please use only ONE of the following measures when providing your answer: either grams, kilograms, pounds or tons)

grams _____

kilograms _____

pounds ______

tons _______

Please read the following question and answer in the space below. Please ONLY use your intuition and not external sources of information. How much calories do you think are contained in 1 gallon of standard dairy milk?

calories_______

I find environmental and ecological issues:

|  | 1 | 2 | 3 | 4 | 5 |
| --- | --- | --- | --- | --- | --- |
| not important🡪important |  |  |  |  |  |
| not essential🡪essential |  |  |  |  |  |
| not valuable🡪valuable |  |  |  |  |  |
| not interesting🡪interesting |  |  |  |  |  |
| not significant🡪significant |  |  |  |  |  |

Do you have any educational background in the following areas:

|  | NO background at all | Some background | Highly relevant background |
| --- | --- | --- | --- |
| Chemistry |  |  |  |
| Ecological or environmental studies |  |  |  |
| Physics |  |  |  |

Do you currently own a car?

- Yes (1)
- No (2)

Do you drink dairy milk?

- Yes (1)
- No (2)

What is your age?

What is your gender?

- Male (1)
- Female (2)

What is your approximate household income?

- Less than $25,000 (1)
- $25,001 - $35,000 (2)
- $35,001 - $50,000 (3)
- $50,001 - $75,000 (4)
- $75,001 - $100,000 (5)
- More than $100,000 (6)

**Study 1b**

**Please read the following questions and answer in the spaces below.** **Please ONLY use your intuition and not external sources of information.**


**How much CO_2_ do you think is emitted in the production/consumption of 1 gallon of standard gasoline in your car? ("carbon footprint"; please use only ONE of the following measures when providing your answer:** **either** **grams, kilograms, pounds or tons)**:

grams _____

kilograms _____

pounds ______

tons _______

**Please ONLY use your intuition and not external sources of information.**

**How much CO2 do you think is emitted in the production/consumption of 1 gallon of standard dairy milk? ("carbon footprint"; please use only ONE of the following measures when providing your answer:** **either** **grams, kilograms, pounds or tons)**

grams _____

kilograms _____

pounds ______

tons _______

**Please ONLY use your intuition and not external sources of information.**

**How long is the distance from Los Angeles to New York (coast-to-coast)? (please use only ONE of the following measures when providing your answer:** **either** meters, kilometers, yards or miles**)**

meters _____

kilometers _____

yards ______

miles _______

**Please ONLY use your intuition and not external sources of information.**

**How many calories do you think are contained in 1 gallon of standard dairy milk?**

calories_______

**Please complete the remaining demographic questions:**

**I find environmental and ecological issues:**

|  |  | \| 1 \| 2 \| 3 \| 4 \| 5 \| \| --- \| --- \| --- \| --- \| --- \| | | | | |  |  |  |  |
| --- | --- | --- | --- | --- | --- | --- | --- | --- | --- | --- | --- | --- | --- | --- | --- |
| not important |  |  |  |  |  |  |  |  |  | important |

Your major at the university is:______________________________________

Do you currently own a car? Y/N

Do you drink dairy milk? Y/N

**Study 2**

**Please read the following question and answer in the space below.** **Please DO NOT USE external sources of information – DO NOT OPEN A NEW WINDOW ON YOUR SCREEN – we are NOT INTERESTED in the “right” answer but in YOUR BEST ESTIMATION.**


What quantity of CO2 do you think is emitted by consuming 1 gallon of standard gasoline when driving a motor vehicle? **("carbon footprint")**

pounds ______

**Please read the following question and answer in the space below.** **Please DO NOT USE external sources of information – DO NOT OPEN A NEW WINDOW ON YOUR SCREEN – we are NOT INTERESTED in the “right” answer but in YOUR BEST ESTIMATION.**


What do you think is the weight of an average family car in the U.S.?

pounds ______

**Please read the following question and answer in the space below.** **Please DO NOT USE external sources of information – DO NOT OPEN A NEW WINDOW ON YOUR SCREEN – we are NOT INTERESTED in the “right” answer but in YOUR BEST ESTIMATION.**

**What do you think is the distance between Los Angeles and New York (coast-to-coast)?**

miles ______

**Please read the following question and answer in the space below.** **Please DO NOT USE external sources of information – DO NOT OPEN A NEW WINDOW ON YOUR SCREEN – we are NOT INTERESTED in the “right” answer but in YOUR BEST ESTIMATION.**

How many calories do you think are contained in 1 gallon of whole milk?

calories ______

To verify that you are carefully reading instruction please choose option three in the following question and ignore its content.

How is your health? (1= excellent, 7 = terrible)

**Please complete the remaining questions:**

**I find environmental and ecological issues:**

|  |  | \| 1 \| 2 \| 3 \| 4 \| 5 \| \| --- \| --- \| --- \| --- \| --- \| | | | | |  |  |  |  |
| --- | --- | --- | --- | --- | --- | --- | --- | --- | --- | --- | --- | --- | --- | --- | --- |
| not important at all |  |  |  |  |  |  |  |  |  | very important |

Please refer to your perception of the following (1 = strongly against, 7 = strongly favor):

1. Capital punishment
2. Abortion (prolife)
3. Gun control
4. Socialized health care
5. Same-sex marriage
6. Illegal immigration

In the coming elections who do you intend to vote for:

The Democratic candidate/the Republican candidate/the independent candidate/I will not vote)

What is your gender: M/F

What is your age: _________

What is your level of education:

1. Less than 8 years (b) 8-12 years (c) Bachelor level academic education (d) Master level academic education (e) PhD (f) other: _______________

What is your annual income level:

Less than 30,000 USD

30,000-39,999 USD

40,000-49,999 USD

50,000-59,999 USD

60,000-69,999 USD

70,000-79,999 USD

80,000-89,999 USD

90,000-99,999 USD

100,000 USD or more

Do you currently own a motor vehicle? Y/N

How often do your travel by air?

(1-5): Never, Rarely (once in few years), Somewhat Often (a few times a year), Often (several times each month), Very Often (almost daily/ daily)

How often do you drink dairy milk?

(1-5): Never, Rarely (less than once a week), Somewhat Often (once or twice per week), Often (several times a week), Very Often (almost daily/ daily)

**Methodological Appendix B: Detailed Analysis Information**

**Methodology for Testing for Differences in Estimation Error and Bias**

***Comparison of Estimation Error of CO_2_ with milk calories, distance, and car weight:***

A mixed-effects ANOVA model is used to determine whether there is a true difference in the mean estimation error of CO_2_ from gasoline versus the estimation error of calories in milk, distance in travel, and car weight. The ideal ANOVA model requires that the data exhibit 1) independence of observations 2) normality of residuals after model fitting and 3) equality of variances**.** The ANOVA model is robust to the deviations from those ideal assumptions that occur in the data sets used for the three studies. The following section discusses the deviations and why they do not impact the conclusions of the paper.

**Independence of Observations:** each participant completed the survey on their personal computers and it is unlikely that there are issues with independence. Issues with independence could occur if, for example, two participants were able to share information or if participants did not follow directions and looked up estimates online. **Normality of Residuals:** The p-values generated by the ANOVA model are generated using the F-test. The F-test assumes that the residuals from the model fit are normally distributed asymptotically. This is evaluated by examine the residuals versus fitted data points can be examined for obvious patterns. A pattern indicates that there is a factor that hasn’t been considered and the inference from the F-test is invalid. The log-transformation of the estimation error shows no significant pattern and conclusions from the F-tests are the same as presented in the text (not transformed). Further, violations of normality reduce the power and have a minor impact on the Type I error rate (Glass, 1972). **Equality of Variance:** ANOVA assumes equality of variance of the factors considered. Violation of this assumption reduces the power of ANOVA. In this study, the large sample size allowed detection of an effect despite the reduction in power. Unequal variance can also change the interpretation of F-test results. When the distributions under comparison (i.e., CO_2_, milk calories, distance or car weight) have different variances, comparing the means might give an incorrect conclusion. In the case of estimation error, a larger mean indicates that the estimation error for the entire population is larger (e.g. mean CO_2_ error vs mean milk error), allowing for valid comparisons using the ANOVA model.

The estimation error will be fitted with factor (i.e., CO_2_, milk calories, distance or car weight) as a fixed effect and subject as a random effect. That is:

$$total estimation error =x+\eta+\epsilon$$

where η is random effect due to subject and ϵ is the within-subject random error. Specifying a random effect due to the subject accounts for the paired nature of the data (i.e., that each individual is asked to estimate all of CO_2_, calories, distance and car weight) This ANOVA model is fit for every study.

***Subgroup Analysis for CO_2_ estimation error***

Estimation errors for CO_2_ are also modeled as a function of gender, political affiliation and rated importance of environmental factors (1: not important at all, 5: very important), with respondent again treated as a random effect.

Since this is a post-hoc analysis, the significance p-value of 0.05 is Bonferroni-adjusted. Every additional factor tested increases the chances of finding a p-value of 0.05. To compensate for this inflation a new significance value is calculated by dividing 0.05 by the number of factors considered. In this case 32 different factors are considered so the new p-value for significance is 0.05/32 = 0.00156.

**Study 1b**

|  | Mean | Std.Error | DF | t-value | p-value |
| --- | --- | --- | --- | --- | --- |
| Baseline:env-rating-3 | 1.993 | 0.580 | 96 | 3.435 | 0.001 |
| env-rating-4 | 0.548 | 0.635 | 96 | 0.862 | 0.391 |
| env-rating-5 | 0.268 | 0.615 | 96 | 0.436 | 0.664 |

In study 1b, no respondent had an environmental rating below 3. The ANOVA analysis suggests that there is no effect of environmental importance on estimation error (there is no difference in mean estimation error between different environmental ratings).

**Study 2**

|  | Value | Std. Error | DF | t-value | p-value |
| --- | --- | --- | --- | --- | --- |
| Baseline:male_Republican_env-rating-1 | 0.869 | 0.171 | 951 | 5.078 | 0.000 |
| Female | -0.222 | 0.047 | 951 | -4.741 | 0.000 |
| Age | 0.005 | 0.001 | 951 | 3.129 | 0.002 |
| Democrat | -0.005 | 0.059 | 951 | -0.080 | 0.937 |
| Independent | 0.099 | 0.064 | 951 | 1.552 | 0.121 |
| Other Political Affiliation | 0.037 | 0.117 | 951 | 0.316 | 0.752 |
| env-rating-2 | 0.165 | 0.182 | 951 | 0.905 | 0.366 |
| env-rating-3 | -0.182 | 0.159 | 951 | -1.148 | 0.251 |
| env-rating-4 | -0.087 | 0.156 | 951 | -0.554 | 0.580 |
| env-rating-5 | -0.153 | 0.155 | 951 | -0.990 | 0.323 |

It appears that gender is significant after a Bonferroni adjustment (0.05/32 = 0.00156). Age, political affiliation and environmental importance are not significant.
